# Supplementary material for: Novel sound exposure drives dynamic changes in auditory lateralization that are associated with perceptual learning in zebra finches
Source: Commun Biol. 2023 Nov 27;6:1205. doi: 10.1038/s42003-023-05567-7 (PMC10681987; doi:10.1038/s42003-023-05567-7)
Supplement: Supplementary file 2 — Supplementary Information [file 42003_2023_5567_MOESM2_ESM.pdf]

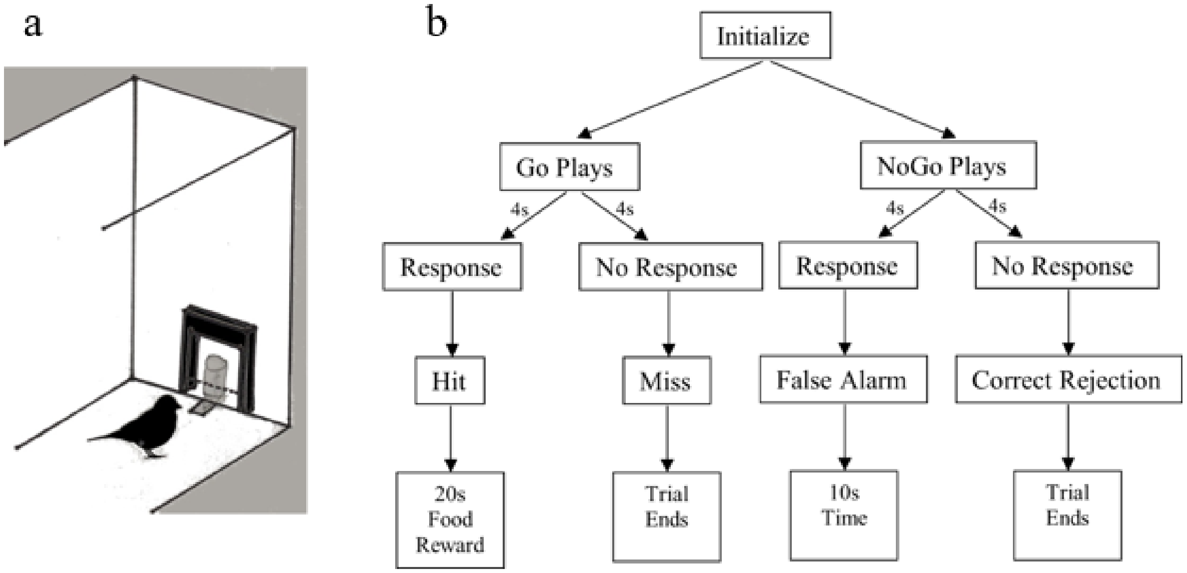

Supplementary Figure 1. Operant Go/NoGo training paradigm. A) Operant training apparatus consisted of a cage with a sensor to detect pecking response and a cutout on the floor of the cage for access to a retractable feeder. B) Flow chart of contingencies for operant training. Subjects were shaped to peck for trial initiation. Once a trial was initiated, a Go or NoGo song (50/50 probability) would be played through the speakers. If a Go song was presented, subjects had 4s to respond with a second peck in order to obtain access to a food reward for 20s. If a NoGo song was played, subjects had to withhold pecking to avoid receiving a 10s lights-out timeout punishment.
