# Supplementary material for: Novel sound exposure drives dynamic changes in auditory lateralization that are associated with perceptual learning in zebra finches
Source: Commun Biol. 2023 Nov 27;6:1205. doi: 10.1038/s42003-023-05567-7 (PMC10681987; doi:10.1038/s42003-023-05567-7)
Supplement: Supplementary file 5 — Reporting Summary [file 42003_2023_5567_MOESM5_ESM.pdf]

nature portfolio

Corresponding author(s): Basilio Furest Cataldo, David Vicario

Last updated by author(s): 10/25/2023

Reporting Summary

Nature Portfolio wishes to improve the reproducibility of the work that we publish. This form provides structure for consistency and transparency in reporting. For further information on Nature Portfolio policies, see our Editorial Policies and the Editorial Policy Checklist.

Statistics

For all statistical analyses, confirm that the following items are present in the figure legend, table legend, main text, or Methods section.

n/a	Confirmed	
☐	☒	The exact sample size (*n*) for each experimental group/condition, given as a discrete number and unit of measurement
☐	☒	A statement on whether measurements were taken from distinct samples or whether the same sample was measured repeatedly
☐	☒	The statistical test(s) used AND whether they are one- or two-sided<br>*Only common tests should be described solely by name; describe more complex techniques in the Methods section.*
☐	☒	A description of all covariates tested
☐	☒	A description of any assumptions or corrections, such as tests of normality and adjustment for multiple comparisons
☐	☒	A full description of the statistical parameters including central tendency (e.g. means) or other basic estimates (e.g. regression coefficient) AND variation (e.g. standard deviation) or associated estimates of uncertainty (e.g. confidence intervals)
☐	☒	For null hypothesis testing, the test statistic (e.g. *F*, *t*, *r*) with confidence intervals, effect sizes, degrees of freedom and *P* value noted<br>*Give P values as exact values whenever suitable.*
☒	☐	For Bayesian analysis, information on the choice of priors and Markov chain Monte Carlo settings
☒	☐	For hierarchical and complex designs, identification of the appropriate level for tests and full reporting of outcomes
☒	☐	Estimates of effect sizes (e.g. Cohen's *d*, Pearson's *r*), indicating how they were calculated

Our web collection on statistics for biologists contains articles on many of the points above.

Software and code

Policy information about availability of computer code

Data collection	Spike2 v7 (CED): custom recording and analysis scripts (shared upon request)<br>MATLAB 2015b (MathWorks): custom code adopted and modified from Gess et al., 2011; was used for behavioral training
Data analysis	Spike2 v7 (CED): custom script for data pre-processing (shared upon request)<br>MatLab 2015b(MathWorks): Multi-unit pre-processing custom-scripts (shared upon request)<br>OriginPro 2020b

For manuscripts utilizing custom algorithms or software that are central to the research but not yet described in published literature, software must be made available to editors and reviewers. We strongly encourage code deposition in a community repository (e.g. GitHub). See the Nature Portfolio guidelines for submitting code & software for further information.

Data

Policy information about availability of data

All manuscripts must include a data availability statement. This statement should provide the following information, where applicable:

- Accession codes, unique identifiers, or web links for publicly available datasets
- A description of any restrictions on data availability
- For clinical datasets or third party data, please ensure that the statement adheres to our policy

Source data for figures and analyses, and datasets acquired from, and preprocessed for, each experiment are available from the corresponding author upon request

Human research participants

Policy information about studies involving human research participants and Sex and Gender in Research.

Reporting on sex and gender	*Use the terms sex (biological attribute) and gender (shaped by social and cultural circumstances) carefully in order to avoid confusing both terms. Indicate if findings apply to only one sex or gender; describe whether sex and gender were considered in study design whether sex and/or gender was determined based on self-reporting or assigned and methods used. Provide in the source data disaggregated sex and gender data where this information has been collected, and consent has been obtained for sharing of individual-level data; provide overall numbers in this Reporting Summary. Please state if this information has not been collected. Report sex- and gender-based analyses where performed, justify reasons for lack of sex- and gender-based analysis.*
Population characteristics	*Describe the covariate-relevant population characteristics of the human research participants (e.g. age, genotypic information, past and current diagnosis and treatment categories). If you filled out the behavioural & social sciences study design questions and have nothing to add here, write "See above."*
Recruitment	*Describe how participants were recruited. Outline any potential self-selection bias or other biases that may be present and how these are likely to impact results.*
Ethics oversight	*Identify the organization(s) that approved the study protocol.*

Note that full information on the approval of the study protocol must also be provided in the manuscript.

Field-specific reporting

Please select the one below that is the best fit for your research. If you are not sure, read the appropriate sections before making your selection.

☒ Life sciences ☐ Behavioural & social sciences ☐ Ecological, evolutionary & environmental sciences

For a reference copy of the document with all sections, see nature.com/documents/nr-reporting-summary-flat.pdf

Life sciences study design

All studies must disclose on these points even when the disclosure is negative.

Sample size	Sample sizes were not calculated for each experiment. Minimal sample size was approximated based on previous studies that assayed the same/similar phenomena in the ZF (e.g. Yang & Vicario, 2015).
Data exclusions	In order to obtain clear event-related potentials (ERPs; Figure A-C) and data that properly assayed lateralized differences in auditory-evoked epidural activity, the longitudinal experiment relied on the chronic epidural array pins, and its grounding wire, to make proper contact with the dura for the full duration of the longitudinal experiment (Figure 2B). One bird from each group of this experiment were dropped from data analysis due to unstable ERPs which were explained by improper positioning of the epidural array.
Replication	The same phenomenon (dynamic shifts in lateralization with HETENVexposure) was tested and confirmed twice using different methodologies: auditory-evoked epidural electrophysiology and acute NCM electrophysiology; while both methods served different purposes in the experiment, (temporal and spatial resolution of phenomenon), they inadvertently served as replications.<br>The effect of silence of lateralized activity was tested twice, with different methods, which served as replications of the same study: one acute(which tested one cohort at one timepoint) and the other chronic-acute (which tested a different cohort twice, at two different timepoints
Randomization	For all experiments, ZFs were randomly sampled from our aviary. For any experiment with 2+ groups, ZFs were further randomly assigned to each group.
Blinding	Data collection was not blinded as any behavioral or electrophysiological assay was performed by the same researchers whom were able to hear which acoustic environment they given ZF was experiencing. The same researchers conducted data analysis and were not blind to this process.

Reporting for specific materials, systems and methods

We require information from authors about some types of materials, experimental systems and methods used in many studies. Here, indicate whether each material, system or method listed is relevant to your study. If you are not sure if a list item applies to your research, read the appropriate section before selecting a response.

Materials & experimental systems

n/a	Involved in the study
☒	☐ Antibodies
☒	☐ Eukaryotic cell lines
☒	☐ Palaeontology and archaeology
☐	☒ Animals and other organisms
☒	☐ Clinical data
☒	☐ Dual use research of concern

Methods

n/a	Involved in the study
☒	☐ ChIP-seq
☒	☐ Flow cytometry
☒	☐ MRI-based neuroimaging

Animals and other research organisms

Policy information about studies involving animals; ARRIVE guidelines recommended for reporting animal research, and Sex and Gender in Research

Laboratory animals	Adult (120+ phd), male Zebra finches (Taeniopygia guttata) were used in all experiments
Wild animals	Wild animals were not used in any of the experiments.
Reporting on sex	Only males were used in this study, sex was confirmed via physiological and behavioral phenotype (e.g. feather patterning, singing). The employment of only males in the current study were informed by the finding of the assayed phenomenon in male ZFs (Yang & Vicario, 2015).
Field-collected samples	Field-collected samples were not used in the current study.
Ethics oversight	All animal care and experimental procedures were employed in accordance to protocol that were approved by the Animal Care and Use Committee at Rutgers University.

Note that full information on the approval of the study protocol must also be provided in the manuscript.
